# Author Index for Volume 97

**DOI:** 10.1038/sj.bjc.6604138

**Published:** 2007-12-11

Aasebø, U 283 Abnet, CC 123, 1567 Abrams, JA 1606 Adam, P 949 Adami, H-O 1570 Adamkova, L 231 Adelman, AS 140 Adigbli, DK 502 Adjadj, DJ 883 Agalliu, I 826 Agapitos, E 1124 Agelaki, S 43 Aguayo, F 85 Ahn, IS 1655 Ahn, JB 458 Ahn, JS 22 Ahn, MJ 22 Ahn, Y-C 22 Aikou, T 405 Aitini, E 1613 Akaike, H 494 Akiba, S 85 Akiyama, F 654 Alami, N 58 Al-Batran, S-E 177 Alderden, RA 194 Alexandre, J 290 Alfaro, J 1206 Ali, S 761 Allen, J 1225 Allgayer, H 1432 Alonso-González, C 755 Alston, RD 1588 Alves, F 1432 Amado, RG 1469 Amant, F 1194 Amaratunga, G 327 Andera, L 73, 1716 Andersen, TT 327 Anderson, JH 1266 Andersson, S-O 730 Anderton, JA 267 Andrén, O 730 Andres-Beck, B 1322 Andreuccetti, M 1613 Andries, L 1344 Androulakis, N 43 Angibaud, P 1344 Angus, B 29 Ansell, P 1310 Ansell, W 308, 1188 Antoni, G 1046 Antonuzzo, A 1613 Aozasa, K 952 Aramin, N 1432 Araya, T 778 Arcaro, KF 809 Arena, MG 593 Arguin, G 1505 Aridome, K 405 Armesilla, AL 745 Artru, P 297 Arts, J 1344 Arvanitakis, M 582 Asakawa, T 162 Asterling, S 308, 1188 Astone, A 1035 Astudillo, A 201 Atmaca, A 177 Attard, G 1338 Atz, J 315 Atzpodien, J 1329 Aubard, Y 883 Aubert, C 919 Aubert, J-P 345 Audoynaud, C 1218 Auperin, A 1664 Autschbach, F 1271 Auvinen, A 115 Auvrignon, A 238 Averill, J 646 Avery-Kiejda, KA 1225 Aviv, A 1696 Axelsson, O 1583

Baas, P 276 Baas-Thijssen, MCM 717 Bagnardi, V 802 Bahra, M 523 Bailey, A 283 Bain, C 1295 Bajetta, E 1469 Ball, J 1696 Ballardini, M 1499 Bamias, A 1124 Bannon, JH 1234 Baptiste, C 1505 Barbachano, Y 670 Barbieri, R 1613 Bardadin, K 531 Bardin-Mikolajczak, A 832 Barduagni, M 1040 Barker, SE 210 Baron, A 391 Barone, C 1035 Bart, J 322 Bartelink, H 712 Bartlett, JMS 378 Barwell, J 1696 Basso, M 1035 Bast, A 1084 Bates, DO 223 Batra, SK 345 Baumann, H 1513 Bearzi, I 92 Bedke, J 1271 Beebe, K 741 Beer, A 1146 Belanger, S 58 Belderbos, J 276 Belli, F 1040 Benedict, A 152 Benhadji, K 628 Bennett, JA 327 Bennouna, J 297 Benyoub, A 883 Beral, V 434 Berardi, R 92 Bercovich, E 1499 Berg, J 1696 Berger, MR 1106 Bergkvist, L 105 Bergström, A 429 Berkhof, J 1084 Berney, D 1188 Berteloot, P 1194 Bertheau, P 1157 Bertholon, J 1218 Betts, G 1017 Birch, JM 1588 Birnboim, HC 1242 Bishop, DT 1701 Bishop, H 725 Bismar, TA 730 Bissett, D 844 Blagden, SP 1338 Blanc, J-F 862 Blanquicett, C 919 Bleijenberg, G 612 Bobrow, M 486 Bodempudi, V 513 Bodoky, G 1469 Body, JJ 964 Boehm, M 523 Bogdanova, T 818 Bogdanovic, G 992 Bohic, S 194 Boige, V 862 Bole, C 14 Bollet, MA 1046 Bonezzi, K 888 Bonnier, P 883 Boot, H 712 Borbath, I 582 Borrow, R 989 Bosetti, C 995 Bottiglieri, L 802 Bottomley, A 302 Bouché, O 862 Bouchet, BP 1218 Boudny, V 231 Bourdon, J-C 277 Bourhis, J 1664 Bozec, A 65 Bozionelou, V 43 Braly, PS 1618 Bray, D 670 Bremnes, RM 283 Brenner, H 1486, 1597 Breuzard, G 562 Brewster, DH 999 Briasoulis, E 1560 Bridle, H 247 Brinton, LA 832 Brisse, H 238 Broderick, P 1305 Bronzwaer, JGF 1084 Brown, EJ 1189 Brown, TJ 358 Brücher, BLDM 1404 Bruynzeel, AME 1084 Buffler, PA 1315 Bugat, R 297, 857 Bukawa, H 792 Bulliard, J-L 1013 Burkholder, I 1480 Burton, C 1310 Buse, S 1271 Butler, P 1601 Butow, PN 6 Büchler, MW 598 Buxton, ILO 1372 Byrne, P 1333

Cafaro, V 1354 Cairncross, JG 302 Cal, S 201 Calgiuri, M 1598 Callaghan, R 194 Calvo, F 628 Calvo, JC 1683 Cameron, DA 479 Campbell, C 1601 Campbell, RB 910 Camplejohn, R 1696 Campo, L 1277 Camponovo, A 1139 Campos, S 1618 Capetillo, M 85 Carén, H 1416 Carles, J 1206 Carlsen, K 1020 Carmi, VD 1655 Carmon, L 1655 Carpenter, B 576 Carter, N 1696 Casas, G 1683 Cascinu, S 92 Casey, G 557 Cassano, A 1035 Cassidy, J 745 Castellone, MD 1545 Castera, D 1200 Castillo, A 85 Catalan, G 1206 Cats, A 712 Cavalli, F 1139 Cave, J 472 Celetti, A 1545 Celsing, F 769 Cen, L 785 Chakrabarti, M 1266 Chan, AT 1295 Chan, FKL 895 Chan, JK 605 Chan, K-K 1409 Chan, K-W 1409 Chan, S 1618 Chan, Y-P 1409 Chandler, I 1305 Chang, JS 1315 Chang, J-Y 334 Chang-Claude, J 1486 Chanock, SJ 832 Chanudet, E 949 Chapelin, D 1157 Chatelut, E 290 Chaturvedi, P 345 Chaudhuri, K 574 Chauvenet, L 1200 Chen, C-C 334 Chen, C-S 785 Chen, G-s 420 Chen, J 1577 Chen, L-T 334 Chen, W 123 Cheng, I 557 Cheng, Y 1441 Cheng, YY 895 Cheung, MK 605 Chevrette, M 941 Chiao, J 628 Chien, C-R 275 Chilvers, C 434 Choi, IJ 700 Choi, KS 700 Choi, YS 22 Chou, W-C 877 Choudhury, A 105, 769 Chrousos, GP 637 Chu, Y-Y 1409 Chung, HC 458 Ciccolini, J 919 Clarke, SJ 1071 Clavère, P 883 Clézardin, P 964 Clements, K 725 Clifford, SC 267 Cnattingius, S 1583 Cochrane, BB 98 Coens, C 302 Coffey, J 1701 Cognettti, F 1015 Cohn, D 605 Colditz, GA 1295 Coleman, D 1601 Coleman, MP 999, 1009 Colleoni, M 802 Colman, L 14 Comandini, D 1469 Conte, P 1469 Cooke, TG 1632 Cooper, M 844 Cooper, R 1333 Coppes, RP 322 Corbishley, C 670 Corte, MD 957 Corvalan, A 85 Cos, S 755 Costanzo, ES 1625 Cotton, SC 133 Coudert, B 857 Coudry, RA 678 Couturier, J 238 Cowan, C 705 Craven, I 1333 Crellin, A 1333 Crnković-Mertens, I 1271 Crossley, B 434 Crouch, S 1310 Crozier, JEM 1266 Cruickshank, ME 133 Culine, S 857 Cunha, IW 678 Cvitkovic, E 628 Czarnocka, B 531

D'Alessandro, C 802 D'Alessio, G 1354 D'Argento, E 1035 d'Enghien, CD 238 D'Ottavio, AM 1040 Dacre, J 472 Dahan, L 862 Daisaki, H 1493 Dalton, P 670 Dalton, SO 1020 Darling, JL 745 Das, T 1361 David, O 1552 Davies, ML 384 Davies, T 464 Daw, NC 1552 Dawsey, SM 123, 1567 Day, JM 1673 de Bono, JS 1338 de Groot, DJA 1077 de Jong, S 1077 de Jonge, M 1338 De Knijf, A 1194 De Lorenzo, C 1354 de Roquancourt, A 1157 De Rosa, G 1545 de Thé, H 1157 de Vries, EGE 322, 1077 de Witte, O 302 Debled, M 1642 DeFreest, LA 327 Delattre, O 238 Delavault, P 253 Dellapasqua, S 802 Delord, J-P 297 Delys, L 818 Denkert, C 523 Derkow, K 769 Desseigne, F 297 Detours, V 818 Deutsch, E 1664 Devercelli, G 1469 Dewar, J 725 Dewhirst, MW 735 Di Benedetto, M 1545 Di Lauro, L 593 Diamond, A 826 Dietel, M 523 Dillner, J 129 DiMarsico, R 1613 Dimopoulos, M-A 1124 Dirix, L 1165 Dirix, LY 659 Discalzi, G 1300 Dittrich, C 1480 Divitini, M 686 Dixon, JM 1182 Do, M-S 1655 Docherty, Z 1696 Dodwell, D 725, 1211 Domenech, M 1206 Donamaria, C 1642 Dong, Z-W 123, 1567 Doriath, V 964 Douillard, JY 297 Douillard, J-Y 857 Drabik, G 589 Du, M-Q 949 Duan, D 1441 Duan, R-D 1441 Dubbelman, R 712 Ducreux, M 862 Dudakia, D 1701 Dudek, AZ 513 Dufer, J 562 Dumont, JE 818 Dunn, DBA 223 Dupouy, N 862 Durand, M 1642 Düring, M 1020

Easton, DF 1701 Eccles, S 670 Eden, TOB 1588 Edler, L 1480 Edwards, J 378 Efficace, F 302 Eijkemans, MJC 868 Eisen, T 247 Eisenbach, L 1655 Eizuru, Y 85 Ejeskär, K 1416 el Galta, R 247 El-Khoury, V 562 Elliott, F 1701 Ellison, DW 267 Elomaa, I 1028 Emery, J 486 Encioiu, E 1690 Enders, GA 1388 Ernst, T 1475 Errami, A 1457 Espié, M 1157 Esposito, I 1106 Etienne, LL 1139 Evans, TRJ 745

Faivre, S 628 Falcone, A 1613 Falette, N 1218 Fall, K 730 Fallowfield, L 152 Fan, ST 50 Fanciullino, R 919 Fanshawe, T 486 Farooqui, M 1523 Farooqui, N 502 Faroux, R 297 Farrar, WL 1116 Fear, NT 1315 Fegan, C 253 Feingold, D 1606 Felici, A 1015 Fellinger, K 315 Ferretti, G 1015 Feskanich, D 1295 Feugeas, J-P 1157 Fichtner, I 1234 Figueroa-Moseley, C 14 Fiori, M 1499 Fizazi, K 857 Floquet, A 1642 Floren, W 1344 Fluck, M 1329 Foggi, P 593 Fojtova, M 231 Foley, J 183 Fong, PC 1338 Fonseca, FP 678 Font, A 1206 Fontana, A 1613 Fontana, E 1613 Forman, D 1211, 1393 Formento, P 65 Forsyth, P 302 Foster, PA 1673 Fountzilas, G 637 Fourquet, A 1046 Fourré, N 562 Fr Brunsvig, P 283 Franc, B 818 François, E 297 Franceschi, S 995 Francque, S 582 Fransson, S 1416 Frapolli, R 888 Frascogna, V 1664 Frassineti, GL 1499 Frattini, M 1139 Fraumeni, JF 1577 Freshney, RI 1714 Fridkin, M 1655 Friess, H 1106 Fritschi, L 686 Frost, A 1480 Fuchs, CS 98 Fujii, H 494 Fujimura, M 778 Fujita, H 1425 Fujita, N 1399 Fujita, Y 426 Fukushima, M 37 Fukuta, N 1532 Furnival, C 1251 Fushimi, K 792

Gakiopoulou, H 1124 Gallardo, E 1206 Galli, C 1613 Galli, L 1613 Galli, V 1545 Gallimore, A 1017 Galmarini, CM 628, 1218 Gamucci, T 1040 Gao, Y-T 1577 García-Muñiz, JL 957 García-Suárez, O 201 Garcia-Closas, M 832 Gardner, J 1696 Garnero, P 964 Garpestad, OK 283 Gatenby, RA 646 Gatter, KC 1277 Gauffin, F 992 Gautier, H 1200 Geerts, T 1344 Gekeler, V 523 Genet, D 883 Georgekutty, J 327 Georgiou, A 1696 Georgoulias, V 43, 1560 Geraci, M 1588 Ghisini, R 802 Ghisletta, M 1139 Ghoul, A 628 Giacometti, S 919 Giassas, S 43 Giavazzi, R 888 Gielissen, MFM 612 Giese, T 1106 Gilbertson, RJ 267 Gillessen, S 1480 Gillies, RJ 646 Gilmore, JL 183 Giovannini, N 1499 Giovannucci, EL 98 Gires, O 315 Giuliante, F 1035 Givalos, N 1124 Glatstein, E 452 Gnad-Vogt, U 1475 Gnant, M 1021 Górka, B 531 Godkin, A 1017 Goehrig, D 964 Goldbohm, RA 1291 Goldhirsch, A 802 Goldie, SJ 1322 Goldstein, D 464 Goldwasser, F 290 Gonterman, RM 183 González, A 755 González, LO 957 González, MV 201 González-Larriba, JL 1206 Gonzalez del Alba, A 1206 Goodchild, R 486 Gooding, LR 992 Gore, ME 1618 Goteri, G 949 Göttlicher, M 177 Gozgit, JM 809 Graf, MR 619 Grann, VR 1606 Granstam-Björneklett, H 105 Gray, R 1305 Green, SR 628 Gregory, CA 1552 Greil, R 1021 Gretz, N 1432 Greving, JP 429 Grieshaber, CK 58 Griffin, RJ 513, 1523 Groen, HJM 322 Grosse, SM 210 Gröne, EF 1271 Groves, FD 140 Grützmann, R 1432 Guarino, V 1545 Guastalla, J-P 1200 Guichard, F 1200 Gunelli, R 1499 Guo, X 745 Gupta, K 1523 Gurda, A 589 Gustafsson, B 992

Haddad-Guichard, Z 1200 Haferkamp, A 1271 Hagiwara, S 1532 Haglund, B 1583 Haim, EB 1655 Hale, GA 539 Hall, MD 194 Hamaguchi, T 170 Hamamura, RS 1099 Hamashima, C 1493 Hambley, TW 194 Han, J-H 22 Han, T-Q 1577 Hanauske, A-R 1480 Handgretinger, R 539 Hansson, BG 129 Harada, K 1425 Harada, M 1648 Haraguchi, N 543 Harper, SJ 223 Harrington, KJ 670 Harris, AL 1277 Hart, SL 210 Hartley, JA 253, 927 Harvey, J 464 Harvey, JR 761 Hasumi, K 1058 Haug, U 1597 Hautmann, R 1146 Hawinkels, LJAC 398 Hawkins, MM 695 Hayakawa, S 1532 Hebbel, RP 513 Heidrich, B 1432 Heinzel, T 177 Helander, I 105 Helbekkmo, N 283 Henderson, BW 1513 Hendrikse, NH 322 Henken, FE 1457 Hentsch, B 177 Herbst, F 1021 Hermon, C 434 Hersey, P 1225 Hershman, DL 1606 Hertervig, E 1441 Hickok, JT 14 Higashi, M 85 Higo, M 792 Hilakivi-Clarke, L 1570 Hildebrand, D 1146 Hirakawa, K 550 Hiraoka, S 1425 Hirata, K 851 Hjelde, HH 283 Ho, CSK 1251 Ho, JWY 50 Ho, YT 1673 Hochhaus, A 1475, 1480 Hodgson, S 1696 Hoel, DG 140 Hofbauer, F 1021 Hoffmeister, M 1486 Hofheinz, R-D 1475 Hofman, P 65 Hofmann, H-P 523 Hohenfellner, M 1271 Holladay, MS 539 Holmen, J 112 Homma, S 1053 Hommes, DW 398 Hong, L 420 Hoover, RN 688 Hoppe-Seyler, F 1271 Hoppe-Seyler, K 1271 Horgan, K 1211 Horgan, PG 1266 Horii, J 1425 Horii, R 654 Horwitz, EM 1552 Houbiers, G 582 Houédé, N 1642 Houlston, R 1305 Houlston, RS 247, 1449 Hövelmann, S 177 Hoye, NA 29 Hsieh, AC 453 Hsieh, F-C 785 Hsieh, H-P 334 Hsing, AW 1577 Hu, C-Y 877 Huang, W 992 Hubner, RA 1449 Huddart, RA 1701 Hulsey, TC 140 Humblet, Y 1469 Huon, I 238 Hurst, NG 971 Hurt, EM 1116 Husain, AN 368 Husseini, F 297 Hutchison, C 705 Hutson, A 1513

Iizuka, N 1399 Ikeda, M 170 Imoto, I 260 Imre, G 523 Inazawa, J 260 Innes, KE 688 Ino, K 1538 Inoue, H 543 Inoue, M 446 Iovine, R 1545 Ishigami, T 792 Ishihara, Y 1648 Ishitsuka, H 1399 Ishiwata, R 37 Ismail, T 971 Ito, M 260 Ito, N 260 Ito, Y 654 Itoh, K 1648 Itoh, T 85 Iwasaki, LM 826 Iwasaki, M 446 Iwase, T 654 Iyengar, R 539

Jack, A 1310 Jack, W 479 Jacob, J 523 Jacobson, HI 327 Jacobson, JS 1606 Jagadeeswaran, R 368 Jagoditsch, M 1021 Jain, M 118, 1600 Jakesz, R 1021 Janicot, M 1344 Janin, A 1157 Jansen, EPM 712 Janssen, L 1344 Janssens, B 1344 Jaramillo, M 1361 Jarkovsky, J 231 Jasinski, P 513 Jäger, D 598 Jäger, E 177 Jäger, M 315 Javed, MA 1372 Jenkinson, HC 695 Jeschke, U 637 Jesnowski, R 1106 Jeung, H-C 458 Jia, W 619 Jiang, X 1106 Jin, VX 895 Jit, M 989 Joel, SP 308, 1188 Joensuu, H 1028 Johansen, C 1020 Johansson, J-E 730 Johnson, DL 1344 Jolly, KW 1315 Jondal, M 769 Jones, A 472 Jones, D 152 Jones, GL 29 Jones, RB 391 Journe, F 964 Jouve, J-L 862 Juin, P 883 Jung, KW 700 Jung, M 1344 Junquera, S 957 Juste, S 58

Kabat, GC 118, 1600 Kadowaki, T 260 Kajiyama, H 1538 Kakugawa, Y 1493 Kalantaridou, SN 637 Kalikaki, A 1560 Kalra, AV 910 Kalykaki, A 43 Kamangar, F 1567 Kameoka, S 1277 Kaminsky, M-C 857 Kamoto, T 260 Kanazumi, N 1260 Kaniwa, N 145 Kapp, DS 605 Karlins, E 826 Karlsson, M 730 Karnon, J 479 Kasahara, K 778 Kasprzyk, PG 1673 Kasuya, H 1260 Kato, BS 1696 Kato, J 851, 1425 Kato, K 170, 1260 Kato, T 1277 Kato, Y 792 Katsuragi, K 550 Kawaguchi, Y 494 Kawanishi, H 260 Kaye, S 1618 Kayed, H 1106 Ke, Y 218 Kearins, O 725 Keh, C 971 Keleg, S 1106 Kentepozidis, N 43 Kerr, GR 479 Kesterton, I 1696 Khanna, RC 14 Kieler, H 1583 Kikkawa, F 1538 Kim, J 22 Kim, JJ 1322 Kim, K 22 Kim, ST 22 Kim, YS 741 Kimura, H 778 Kimura, M 1532 Kimura, T 1277 King, P 1344 Kinmonth, AL 486 Kinnon, C 210 Kinoshita, T 1260 Kirby, JA 761 Kitazawa, S 1707 Kitzen, J 1338 Kiyosawa, K 145 Kleeff, J 1106 Klijanienko, J 238 Klotz, LH 1690 Knuiman, M 686 Knuth, A 177 Kobayashi, C 1099 Kobayashi, M 1277 Kogner, P 1416 Koizumi, F 778 Kolodziejczyk, P 589 Kolonel, LN 440 Koltz, PF 183 Komohara, Y 1648 Konger, RL 183 Kono, K 494 Kopp-Schneider, A 1146 Korbelik, M 1381 Koriyama, C 85 Korkolopoulou, P 1124 Korpela, R 1028 Koshiol, J 1567 Kotani, K 426 Kotasek, D 464 Kothonidis, K 73, 1716 Kouri, M 1028 Koutsopoulos, A 1560 Kouzu, Y 792 Kovarik, A 231 Kovarik, J 231 Kozu, T 1493 Krasner, CN 1618 Kripp, M 1475 Kristiansen, G 523 Kritz, A 210 Krumroy, LM 557 Krupa, K 1632 Kubben, FJGM 398 Kudo, M 1532 Kudoh, S 162 Kuhn, A 1146 Kuhn, E 888 Kuipers, EJ 868 Kulig, J 589 Kunikane, H 162 Kunitoh, H 162 Kuo, C-C 334 Kurahara, H 405 Kurashina, R 1099 Kurdistani, SK 1 Kuroiwa, G 851 Kurozawa, Y 426 Kut, C 978 Kutner, R 1552 Kwon, EM 826

La Vecchia, C 1300 Labourey, JL 883 Lacoste, J 941 Ladekarl, M 1135 Lagiou, P 1570 Lala, PK 1090 Lamblot, C 1218 Lamelas, ML 957 Lamers, CBHW 398 Landi, L 1613 Langlet, P 582 Langrehr, J 523 Lansdown, M 1180 Laplanche, A 857 Larsson, SC 1005 Lassalle, S 65 Lauerova, L 231 Laughner, R 183 Law, SY-K 1409 Law, WE 1372 Lawrence, G 725 Lawson, AB 140 Le François, BG 1242 Le, TKP 1077 Le Tourneau, C 628 Lebrun-Ly, V 883 Lecluse, Y 1664 Ledermann, JA 927 Leduc, B 1200 Lee, AK 700 Lee, C-Y 877 Lee, H-S 22 Lee, J 22 Lee, JE 429 Lee, K-S 22 Lee, M-J 741 Lee, N 1552 Lee, SY 700 Lee, TK 50 Leek, RD 1277 Leese, MP 1673 Legrès, L 1157 Leitch, EF 1266 Lejeune, C 883 Lekander, M 105 Lemanski, N 327 Lemay, R 1505 Lennard, TWJ 761 Lennette, ET 1567 Lepillé, D 1200 Lesimple, T 857 Leunen, K 1194 Leung, W 539 Leung, WK 895 Levi, F 995, 1013 Levidou, G 1124 Lewis, DA 183 Leyland-Jones, B 58 Li, H 420 Li, J 895 Li, KM 1071 Li, L 1432 Li, Q 420 Li, Y 1523 Lianes, P 1206 Libert, F 818 Lightfoot, T 986 Lin, H-J 785 Lin, J 785 Lin, L-I 877 Lindblad, P 429 Lindblom, A 1175 Lindemalm, C 105 Lindgren, S 1441 Lindhofer, H 315 Lindsey, JC 267 Lippens, C 65 Lissowska, J 832 Little, J 133 Liu, CL 50 Liu, J-F 334, 1449 Liu, S 308, 1188 Liu, X 557 Lo, CM 50 López, JA 1251 Loblaw, DA 1690 Locker, G 152 Logan, RFA 1449 Loizidou, M 502 Looijenga, LH 1707 Lopez, M 593 Lorch, G 183 Loria, RM 619 Lortholary, A 857 Lotze, MT 1598 Lou, L 934, 1715 Löf, M 1570 Löhr, M 1106 Lowe, H 253 Lu, YJ 1707 Luangdilok, S 670 Lubbe, S 1305 Lucenteforte, E 995 Lucraft, H 29 Lugassy, G 1655 Luini, A 802 Lukan, N 1475 Lukkien, C 429 Lusher, ME 267 Lutgendorf, SK 1625 Lynch, J 1020 Lynge, E 1019

Ma, J 98 Ma, PC 368 Mac Gabhann, F 978 Macher-Göppinger, S 1271 Machlenkin, A 1655 MacRobert, AJ 502 Madranges, N 1642 Mady, S 670 Maeda, S 405 Maemura, K 405 Maenhaut, C 818 Magni, E 802 Magnolfi, E 1040 Magnusson, C 1287 Maini, PK 646 Makrigiannakis, A 637 Man, EPS 895 Man, K 50 Mandolesi, A 92 Mansel, R 152 Manson, JE 98 Maraqa, L 1180 Marchetti, S 577 Marconi, SA 809 Margolin, S 1175 Mariën, A 1344 Marijnen, CAM 717 Mark, SD 123, 1567 Marlow, LAV 691 Marosi, C 302 Maroun, JA 1242 Marsden, HB 695 Marselos, M 43 Martínez-Campa, C 755 Martin, C 1200 Martin, J 883 Martin, JE 412 Martin, V 1139 Martinelli, M 888 Martinsson, T 1416 Martinussen, N 986 Mascolo, M 1545 Maspoli, M 1013 Massard, C 857 Masson, LF 133 Mataki, Y 405 Matakidou, A 247 Matsui, Y 260 Matsumoto, K 1053 Matsumoto, S 37 Matsumoto, T 543 Matsumura, Y 170 Matsunaga, T 851 Mattes, ML 1625 Mauer, M 302 Maughan, NJ 1393 Maurer, A 177 Mauriac, L 1642 Märten, A 598 Mavroudis, D 43, 1560 Maxwell, T 1251 Mazza, O 1683 Mazzucchelli, L 1139 Mc Gee, MM 1234 McGlynn, LM 378 McGuckin, MA 1251 McIntyre, A 1707 McKee, RF 1266 McMahon, T 705 McMeekin, DS 1618 McMillan, DC 1266 McPherson, K 434 Mediavilla, MD 755 Meijer, CJLM 1457 Meiss, R 1683 Melcher, A 1333 Melia, J 1601 Meliffi, L 1040 Melillo, RM 1545 Mellado, B 1206 Mellor, HR 194 Mellor, P 761 Mellstedt, H 105 Mercier, C 919 Merino, AM 957 Merolla, F 1545 Merrouche, Y 857 Merx, K 1475 Metayer, C 1315 Meza, JL 345 Mhaidat, NM 1225 Mi, Y 934, 1715 Michael, M 464 Michaud, DS 98 Michon, J 238 Mikaelsson, E 105 Mikeljevic, JS 1211 Mikula, M 531 Milano, G 65 Milbauer, L 513 Miller, AB 118, 1600 Miller, E 989 Mimori, K 543 Mimura, K 494 Minami, R 1053 Minas, V 637 Mirimanoff, RO 302 Misset, J-L 1157 Missiaglia, E 1707 Mitrou, S 637 Mitsui, F 494 Miura, T 1399 Miyamoto, K 778 Miyanishi, K 851 Miyoshi, E 1538 Moasser, MM 453 Modok, S 194 Moerman, P 1194 Molinari, F 1139 Moniaux, N 345 Monroe, KR 440 Montella, M 995 Montgomery, DA 1182, 1632 Moran, A 1588 Morant, R 1480 Mori, M 543 Moribe, T 1399 Moriyama, N 1493 Morizane, C 170 Morris, E 1393 Morris, H 486 Morrow, GR 14 Mortensen, PB 1020 Moscetti, L 1040 Moss, S 1601 Mosseri, V 238 Motta, S 1545 Mould, TA 927 Mozaffari, F 105 Mross, K 1480 Mucci, LA 730 Muir, KR 1449 Mukherjee, R 378 Munakata, H 1532 Munzer, C 238 Muramatsu, Y 1493 Muro, K 170 Murphy, KJ 761 Murphy, SP 440 Murray, GI 576 Murray, S 1560 Murthy, BL 1211 Mutsvangwa, K 308, 1188 Mühlradt, PF 598 Müller-Hermelink, HK 949

Nadella, KS 183 Nagai, Y 37 Nagasaka, T 1538 Nagashima, H 851 Nagashima, M 1532 Nagengast, WB 322 Nagle, RB 646 Naito, M 426, 1648 Nakahama, H 170 Nakai, Y 952 Nakamura, Y 543 Nakao, A 1260 Nakao, S 778 Nakashima, D 792 Nakatani, T 1532 Nakayama, M 952 Nallasura, V 368 Nam, RK 1690 Naman, H 297 Nanni, O 1499 Narendran, A 327 Narod, SA 1187, 1690 Nasioulas, G 73, 1716 Natsugoe, S 405 Naucler, P 129 Navone, N 1683 Nawa, A 1538 Nawaz, F 391 Nawaz, S 391 Neal, K 133 Neckers, L 741 Nedergaard, BS 1135 Neff, JK 1432 Negri, E 995, 1300 Neill, M 1690 Neugut, AI 1606 Neuhaus, P 523 Neumann, A 177 Neven, P 1194 Newman, SP 1673 Newton, C 927 Newton, R 1310 Ng, I 50 Ng, KT 50 Nicholson, AG 949 Nie, L 420 Niedergethmann, M 1432 Nielsen, K 1135 Niesporek, S 523 Niessen, HWM 1084 Nieto, Y 391 Niitsu, Y 851 Nikolaidou, M 43 Nilsen, TIL 112 Nilsson, Å 1441 Nilsson, B 105 Ning, T 218 Nishikawa, T 1277 Nishimura, K 952 Nishio, K 426, 778 Nishiyama, H 260 Niwa, S 1577 Noguchi, M 1648 Nogué, M 1206 Noh, SH 458 Noma, H 405 Nomoto, S 1260 Nomura, H 792 Nomura, S 1538 Nonomura, N 952 Norberg, M 769 Nordgren, A 992 Nowicki, M 412 Nunziata, C 593 Nutting, CM 670 Nuzzo, G 1035 Nyengaard, JR 1135 Nygren, AOH 1457

O'Boyle, G 761 Ochi, H 1053 Odutoye, T 670 Ogawa, O 260 Ogawara, K 792 Ogimoto, I 426 Ohira, M 550 Ohnuma, H 851 Ohyashiki, JH 1099 Ohyashiki, K 1099 Oikonomou, E 73, 1716 Ojutkangas, M-L 105 Oka, D 952 Oka, M 1399 Okada, S 1053 Okamoto, H 162 Okamoto, T 851 Oki, A 1053 Okusaka, T 170 Okuyama, A 952 Olaitan, A 927 Oliver, RTD 308, 1188 Ollus, A 1028 Olson, S 1552 Olsson, A 769 O'Neill, A 1234 Ono, K 792 Oosterhuis, JW 1707 Orfeuvre, H 1200 Orlando, A 223 Ornelles, DA 992 O'Rourke, K 140 Osaka, H 550 Osann, K 605 Osoba, D 302 Osorio, LM 769 Ostrander, EA 826 Ota, T 1058 Otani, S 1260 Otani, T 446 Otis, CN 809 Ott, N 1404 Ökvist, A 769 Österborg, A 105, 769 Österlund, P 1028 Overmeer, RM 1457

Paliczka, E 531 Pallis, AG 1560 Pampillón, C 1234 Pan, W-Y 334 Pan, Y 218 Panchapakesa, V 1361 Pandeya, N 1295 Pangon, L 1696 Papaemmanuil, E 1305 Papo, NL 479 Pappagallo, GL 1499 Pappas, P 43 Paquette, B 1505 Paraiso, D 1200 Paraskevaidis, EA 637 Paret, C 1146 Park, EC 700 Park, K 22, 700 Park, KGM 902 Parker, E 1701 Parker, R 1601 Pasquini, E 1499 Paterson, J 58 Patnick, J 1601 Patsouris, E 1124 Paul, J 705 Pavlidis, N 637 Paximadas, D 1696 Paz, A 1655 Pebody, R 989 Peeters, M 582, 1469 Peinado, JR 201 Pelucchi, C 995, 1300 Penegar, S 1305 Peng, B 1116 Pentecost, BT 809 Pentland, AP 14 Peplonska, B 832 Pepper, C 253 Perrier, H 297 Peto, J 434, 1305 Petsas, G 637 Pezzella, F 1277 Pfeifer, S 1583 Piana, L 883 Piccerelle, P 919 Picelli, S 1175 Pidal, I 957 Piedbois, P 297 Pierantoni, C 92 Pierga, J-Y 1046 Pieterse, AH 717 Pignon, J-P 862 Pike, MC 434, 440 Pilarsky, C 1432 Pileri, SA 949 Pintzas, A 73, 1716 Pinzon-Charry, A 1251 Piolatto, PG 1300 Piperi, C 1124 Pira, E 1300 Pirie, K 434 Pitkin, L 670 Pituch-Noworolska, A 589 Pivot, X 857 Plantaz, D 238 Plassa, L-F 1157 Plouffe, B 1505 Plummer, SJ 557 Pollak, MN 98 Ponce, AM 735 Poon, RTP 50 Poonawala, T 1523 Popel, AS 978 Popiela, T 589 Post, S 1432 Potter, BVL 1673 Potts, HWW 472 Powles, T 308, 1188 Pozzo, C 1035 Prevost, AT 486 Priou, F 857 Pritchard-Jones, RO 223 Pritsch, M 1271 Probert, E 1716 Prockop, DJ 1552 Proctor, SJ 29 Pronk, LC 1338 Provencher, DM 1618 Pruneri, G 802 Pugh, JL 1701 Puisieux, A 1218 Puisset, F 290 Pujade-Lauraine, É 1200 Pukkala, E 115 Purohit, A 1673 Pühringer-Oppermann, F 1404 Pye, D 761 Pyke, CM 1251

Qiao, Y-L 123, 1567 Qiu, Y 223 Qualman, SJ 785 Quirke, P 1393

Rabbani, Z 735 Rabl, H 1021 Rachet, B 999 Ranaldi, R 949 Randimbison, L 1013 Rankin, EM 844 Rantala, J 1175 Raobaikady, B 1673 Raoul, J-L 862 Raoul, V 290 Rapley, EA 1701 Rashid, A 1577 Rastogi, S 1090 Raum, E 1486 Ray-Coquard, I 1200 Raymond, E 628 Reece, W 464 Reed, MJ 1673 Regeling, A 1077 Reinier, K 1315 Reiser, J 1552 Reitz, M 1329 Renshaw, FG 1618 Reynaert, H 582 Rha, SY 458 Rhys Evans, P 670 Ribeiro, A 238 Riccardi, E 888 Richesson, D 832 Ricketts-Loriaux, RSJ 809 Rigby, H 223 Ringuette, MJ 358 Risley, P 502 Rivory, LP 1071 Roberts, GT 384 Roberts, H 1277 Roberts, JM 688 Roberts, K 1063 Robertson, R 1601 Robinson, CB 1625 Roché, H 290 Roddam, AW 434 Rodríguez, JC 957 Rodriguez-Sierra, JF 345 Rogers, T 1523 Roh, JK 458 Rohan, TE 98, 118, 1600 Rolaki, A 637 Romagnani, E 1139 Roman, E 986, 1310 Roman, SL 1625 Roose, T 194 Rose, F 646 Rose-John, S 1513 Rosenberg, LU 1287 Rosenquist, R 769 Rosero-Bixby, L 837 Rosol, TJ 183 Ross, L 1020 Roy, P 1361 Roychoudhury, P 574 Rubie, H 238 Rubin, MA 730 Rudd, MF 247 Ruf, P 315 Rumjahn, SM 1372 Ruotsalainen, T 1028 Russo, C 1315 Ruzankina, Y 1189 Ryan, JL 14 Ryd, W 129 Rylander, E 129

Sánchez-Barceló, EJ 755 Sacca, P 1683 Saetta, A 1124 Sagawa, T 851 Saijo, N 162 Saito, D 1493 Saito, H 1493 Saito, K 792 Sakaguchi, K 1425 Sakaguchi, Y 1532 Sakaida, I 1399 Sakamoto, K 1399 Sakoda, LC 1577 Sakurai, M 1053 Salahuddin, FK 735 Salek, T 1469 Saletti, P 1139 Salgia, R 368 Salmon, R 1046 Samonigg, H 1021 Sandin, S 1570 Santoro, M 1545 Sarbia, M 1404 Sasazuki, S 446 Sastre-Garau, X 1046 Sato, Y 851 Satoh, T 1053 Savage, SA 832 Savignoni, A 1046 Sawada, T 550 Saxelin, M 1028 Scartozzi, M 92 Schaberl-Moser, R 1021 Schatzlein, A 745 Scheidereit, C 523 Schellens, JHM 577 Schernhammer, ES 98 Scheulen, M 1480 Schinzari, G 1035 Schippinger, W 1021 Schleiermacher, G 238 Schmidt, C 1251 Schmidt, J 598 Schönthal, AH 1465 Schouten, JP 1457 Schouten, LJ 1291 Schultheis, B 1475 Schüz, J 986 Schwarz, SE 177 Scott, R 194 Scott, RJ 1225 Sebag-Montefiore, D 1333 Seid, PL 605 Selby, P 1063 Sellick, G 1305 Sellick, GS 1449 Senan, S 276 Serova, M 628 Seth, A 1690 Seth, R 133 Shack, LG 999 Shah, A 1009 Shamash, J 308, 1188 Shapiro, J 464 Sharp, L 133 Shastri, YM 1595 Shen, M-C 1577 Shenoy, H 1211 Shepherd, HL 6 Shi, X 218 Shiba, M 952 Shibata, A 426 Shibata, K 1538 Shibata, T 162 Shiiba, M 792 Shim, YM 22 Shimada, Y 170 Shin, HR 700 Shin, JY 605 Shin, SJ 458 Shinchi, H 405 Shipley, J 1707 Shirakusa, T 1648 Shirao, K 170 Shiratori, Y 1425 Shoda, H 1493 Shpall, EJ 391 Shun, K 941 Shuyama, K 85 Siapati, EK 210 Sidiropoulos, J 637 Siena, S 1469 Sier, CFM 398 Sierra, R 837 Siersema, PD 868 Sierzega, M 589 Sigal-Zafrani, B 1046 Sikes, RA 1713 Silvestrini, R 1499 Simpson, J 1310 Sinha, D 140 Skoglund, J 1175 Skubis-Zegadlło, J 531 Smallbone, K 646 Smith, A 1063, 1310 Smith, D 297 Smith, TAD 902 Smolarz, AJ 1552 Snijders, PJF 1457 Soares, FA 678 Sodek, KL 358 Soga, S 741 Sohaib, SA 1701 Soldan, K 989 Soliman, H 1157 Somanath, S 745 Sone, T 778 Song, B 1175 Song, CW 1523 Souckova, K 231 Souglakos, J 1560 Sousi, E 502 Spasojevic, I 735 Spector, TD 1696 Speizer, FE 1295 Sperduti, I 593, 1040 Spry, N 464 Squire, JA 678 Srivastava, G 1409 Srivastava, S 1690 Staibano, S 1545 Stampfer, MJ 98 Stanford, JL 826 Stanimirovic, A 1690 Stålberg, K 1583 Stathopoulos, E 1560 Stebbing, J 308, 1188 Steenbergen, RDM 1457 Steers, G 1277 Steevens, J 1291 Steger, GG 1021 Stein, JM 1595 Steinbild, S 1480 Steiner, J 844 Stevens, MCG 695 Steyerberg, EW 868 Stiggelbout, AM 717 Stiller, CA 695 Stocken, DD 971 Stott, B 1381 Stovall, MA 695 Strand, A 129 Stratton, MR 1701 Strohfeldt, K 1234 Strumberg, D 1480 Stuart, N 384 Stupp, R 302 Suárez, C 201 Sugai, H 494 Sugar, L 1690 Sugimoto, H 1260 Suminoe, M 778 Summersgill, B 1707 Sun, BS 50 Sun, CK 50 Sun, J 1381 Sundstrøm, SH 283 Sung, JJY 895 Sung, NY 700 Surowy, H 1184 Suttie, SA 902 Suzuki, H 426 Sweeney, NJ 1234 Syrigos, KN 670 Szczepanik, A 589 Szeszenia-Da̧browska, N 832

Tabata, T 1058 Tacke, M 1234 Takahashi, N 1538 Takahashi, T 260 Takahashi, Y 851 Takaku, T 1099 Takao, S 405 Takayama, H 952 Takayama, T 851 Takeda, S 1260 Takeshima, N 1058 Takimoto, R 851 Takizawa, K 1058 Talamini, R 995 Talbot-Smith, A 686 Talekar, G 992 Tam, L 378 Tamakoshi, A 426 Tamatsukuri, S 1399 Tamesa, T 1399 Tamori, S 778 Tamura, T 162 Tanaka, F 543 Tanaka, YO 1053 Tanzawa, H 792 Tao, Q 895 Tao, Y 1664 Taoufik, E 1716 Taphoorn, MJB 302 Taraboletti, G 888 Tattersall, MHN 6 Taylor, I 502 Taylor, PR 123, 1567 Taylor, PRA 29 Te, V-C 1013 Tello, JM 1206 Terai, S 1399 Teramukai, S 37 Terauchi, T 1493 Terfloth, K 1329 Testa, D 1545 Tewfik, F 1625 Thaler, J 1021 Theodore, C 857 Therriault, H 1505 Thomas, C 238 Thomas, G 818 Thomas, H 844 Thomas, SB 1116 Thomsen, HF 1135 Thomson, CS 725, 1211 Thödtmann, R 1021 Thrasher, AJ 210 Thurieau, C 253 Thurston, DE 253 Thymara, I 1124 Tien, H-F 877 Timoshenko, AV 1090 Tirosh, B 1655 To, KF 895 Tobin, G 769 Tokuhisa, Y 1399 Toribio, RE 183 Torres, CH 678 Torrisi, R 802 Törnberg, S 129 Trachtenberg, J 1690 Tracy, E 1513 Traynor, P 378 Trehan, S 1625 Treluyer, J-M 290 Trepel, J 741 Tretiakova, MS 368 Trichopoulos, D 112, 1570 Trigila, N 1035 Tripaki, M 1560 Troise, F 1354 Troisi, R 688 Tsai, F-Y 334 Tschmelitsch, J 1021 Tsenga, A 1124 Tsou, T-C 334 Tsugane, S 446 Tsujimoto, G 260 Tsujimura, A 952 Tsunoda, H 1053 Tubiana-Mathieu, N 883 Tuman, RW 1344 Turley, H 1277 Turner, V 539 Turpin, E 1157 Tzehoval, E 1655

Uchida, K 1399 Ueno, H 145, 170 Ulivi, P 1499 Underhill, C 464 Uno, K 1053 Urayama, K 1315 Uzawa, K 792

Vaalburg, W 322 Vázquez, J 957 Vaccaro, A 1040 Vadai, E 1655 Valta, P 1028 Valteau-Couanet, D 238 Vamvakas, L 43 Van Calster, B 1194 Van Cutsem, E 1469 van Dam, P 659, 1165 van de Velde, CJH 717 van den Bent, MJ 302 van den Brandt, PA 1291 Van den Eynden, G 1165 Van den Eynden, GG 659 Van der Auwera, I 659, 1165 van der Deen, M 1077 van der Graaf, WTA 322 van der Hoeven, JJM 1084 van der Vijgh, WJF 1084 van der Zon, JM 398 van Duijn, W 398 van Dun, J 1344 van Emelen, K 1344 van Groeningen, CJ 1084 Van Hazel, G 464, 1469 Van Huffel, S 1194 Van Hummelen, P 1165 Van Laere, S 1165 Van Laere, SJ 659 Van Laethem, J-L 582 Van Marck, E 1165 Van Marck, EA 659 van Rietschoten, JGI 1457 van Vliet, EPM 868 Vandebroek, A 582 Varey, AHR 223 Varna, M 1157 Varshney, GC 345 Vassilopoulos, I 1124 Vatten, LJ 112 Vazquez, E 1683 Vecchia, CL 995, 1013 Velikova, G 1063 Venat-Bouvet, L 883 Ventouri, K 1124 Verborg, W 844 Vergote, I 1194 Verhage, BAJ 1291 Verhagen, CAHHVM 612 Verheij, M 712 Verheijen, JH 398 Vermeulen, PB 659, 1165 Veronesi, P 802 Verset, G 582 Verslype, C 582 Verspaget, HW 398 Verweij, J 1338 Vessey, M 434 Vetter, C 1271 Viale, G 802 Viloria, CG 201 Vincent-Salomon, A 1046 Virtanen, A 115 Vizoso, FJ 957 Vlahovic, G 735 Vogel, W 1184 Voigt, J-J 857 von Euler-Chelpin, M 1019 von Plessen, C 283 Voorzanger-Rousselot, N 964 Voutsina, A 1560 Vujaskovic, Z 735 Vyse, A 989

Wadell, G 129 Wagener, N 1271 Wakai, K 426 Wakelam, MJO 971 Wakeman, JA 384 Waller, J 691 Walpole, E 464 Wang, B-S 1577 Wang, H 420 Wang, J-C 941 Wang, S-f 420 Wang, W 745 Wang, X 1409, 1606 Wang, Y 1513 Wardle, J 691 Wartenberg, D 140 Watanabe, J 260 Watanabe, K 162 Waterfall, J 844 Watson, RW 1234 Waugh, N 133 Webb, EL 247 Wedrén, S 1287 Wegman, TD 322 Wei, L-H 1513 Weichert, W 523 Weiderpass, E 1570 Weiss Solís, D 818 Weitz, J 1146 Weller, C 523 Weller, D 1601 Weller, M 302 Wells, A 1361 Welsch, T 598 Welsh, BW 513 Werelius, B 1175 Whaley, D 1361 Willett, EV 1310 Williamson, P 670 Wilson, DGG 502 Wilson, P 308, 1188 Wilson, POG 670 Wilson, S 971 Wilting, SM 1457 Windebank, KP 29 Winter, DL 695 Winter, J 1486 Winther, JF 986 Witte, JS 557 Wohlmuth, P 1021 Wolf, M 1469 Wolk, A 429, 1005 Wolk, M 412 Wolpin, BM 98 Won, YJ 700 Wong, ML-Y 1409 Wong, N 1372 Wong, R 302 Wong, Y-C 1409 Wong, YP 895 Wood, K 29 Wood, W 1305 Woods, J 391 Woolf, K 472 Woolley, M 1469 Worrall, L 646 Wotherspoon, AC 949 Wright, P 1063 Wu, J 1441 Wyatt, P 308, 1188 Wynne, P 927

Xu, W 741

Yamada, A 1648 Yamada, Y 170 Yamaguchi, K 851 Yamamoto, E 1538 Yamanaka, T 37 Yamano, Y 792 Yamashita, Y 1648 Yanaga, K 543 Yang, L-Y 1690 Yang, W 1690 Yashiro, M 550 Yasui, H 170 Ye, H 949 Yeh, S-C 334 Yin, X 1315 Yokoe, H 792 Yokomine, T 405 Yokoyama, A 162 Yoshikawa, H 1053 Yoshimoto, M 678 Yoshimura, T 426 Yu, C-J 275 Yu, J 895 Yuen, H-F 1409

Zatonski, W 832 Zembala, M 589 Zentgraf, H 1271 Zgonjanin, L 735 Zhang, B-H 1577 Zhang, F 420 Zhang, L-y 420 Zhang, P 1664 Zhang, S 218 Zhang, XD 1225 Zhang, X-q 420 Zhang, Y 1099 Zhao, P 123 Zhi, J 1338 Zhou, X 1175 Zhou, Y 218 Zografos, G 73, 1716 Zoli, W 1499 Zou, L 1361 Zöller, M 1146 Zucchetti, M 888 Zucchetto, A 995 Zugmaier, G 1338 Zuidwijk, K 398 Zvereff, V 941

